# Supplementary material for: Comprehensive Analysis of Kisspeptin Signaling: Effects on Cellular Dynamics in Cervical Cancer
Source: Biomolecules. 2024 Jul 29;14(8):923. doi: 10.3390/biom14080923 (PMC11352469; doi:10.3390/biom14080923)
Supplement: Supplementary file 1 [file biomolecules-14-00923-s001.zip › biomolecules-3040384-supplementary.pdf]

Supplementary Data

	Signaling Pathway	KP10			Ala³-KP10				Ala⁴-KP10			
		**EC_{50}(M)**	**pEC_{50}**	**Emax(%)**	**EC_{50}(M)**	**Fc**	**pEC_{50}**	**Emax(%)**	**EC_{50}(M)**	**Fc**	**pEC_{50}**	**Emax(%)**
Gq Protein Family	G_q	$1.52x10^{-8}$	7.87	100	$8.74x10^{-8}$	5.76	7.07	107.25	$2.23x10^{-7}$	2.55	6.67	103.67
	G_{11}	$4.75x10^{-8}$	7.42	100	$2.21x10^{-7}$	4.65	6.68	102.20	$4.54x10^{-7}$	2.05	6.35	106.42
	G_{14}	$4.12x10^{-8}$	7.48	100	$1.76x10^{-7}$	4.28	6.78	100.16	$3.54x10^{-7}$	2.00	6.45	97.69
	G_{15}	$3.10x10^{-8}$	7.57	100	$1.52x10^{-7}$	4.92	6.87	99.45	$3.60x10^{-7}$	2.36	6.47	100.80
Gi Protein Family	G_{i1}	NQ	NQ	NQ	NQ	NQ	NQ	NQ	NQ	NQ	NQ	NQ
	G_{i2}	NQ	NQ	NQ	NQ	NQ	NQ	NQ	NQ	NQ	NQ	NQ
	G_{i3}	NQ	NQ	NQ	NQ	NQ	NQ	NQ	NQ	NQ	NQ	NQ
	G_{oA}	NQ	NQ	NQ	NQ	NQ	NQ	NQ	NQ	NQ	NQ	NQ
	G_{oB}	NQ	NQ	NQ	NQ	NQ	NQ	NQ	NQ	NQ	NQ	NQ
	G_z	$1.63x10^{-7}$	6.87	100	$6.46x10^{-7}$	3.96	6.24	94.02	$1.10x10^{-6}$	1.70	6.04	84.68
	G_s	NQ	NQ	NQ	NQ	NQ	NQ	NQ	NQ	NQ	NQ	NQ
	G_{12}	NQ	NQ	NQ	NQ	NQ	NQ	NQ	NQ	NQ	NQ	NQ
	G_{13}	NQ	NQ	NQ	NQ	NQ	NQ	NQ	NQ	NQ	NQ	NQ
	β-arrestin1	$5.92x10^{-7}$	6.51	100	$1.19x10^{-6}$	2.01	6.37	69.04	$1.30x10^{-5}$	10.86	5.53	69.09
	β-arrestin2	$3.75x10^{-7}$	6.43	100	$1.43x10^{-6}$	3.82	6.00	63.86	$2.58x10^{-6}$	1.79	5.60	78.99

Table S1: Potency of KP10 and its analogs in activating the differents signaling pathways. EC_{50} and pEC_{50} values are presented as mean from 4 experiments. Fc is the fold change of EC_{50} values for the analogs relative to the EC_{50} value obtained for KP10. Emax was represented as a percentage, with the reference (KP10) being 100%. NQ, Not Quantifiable.

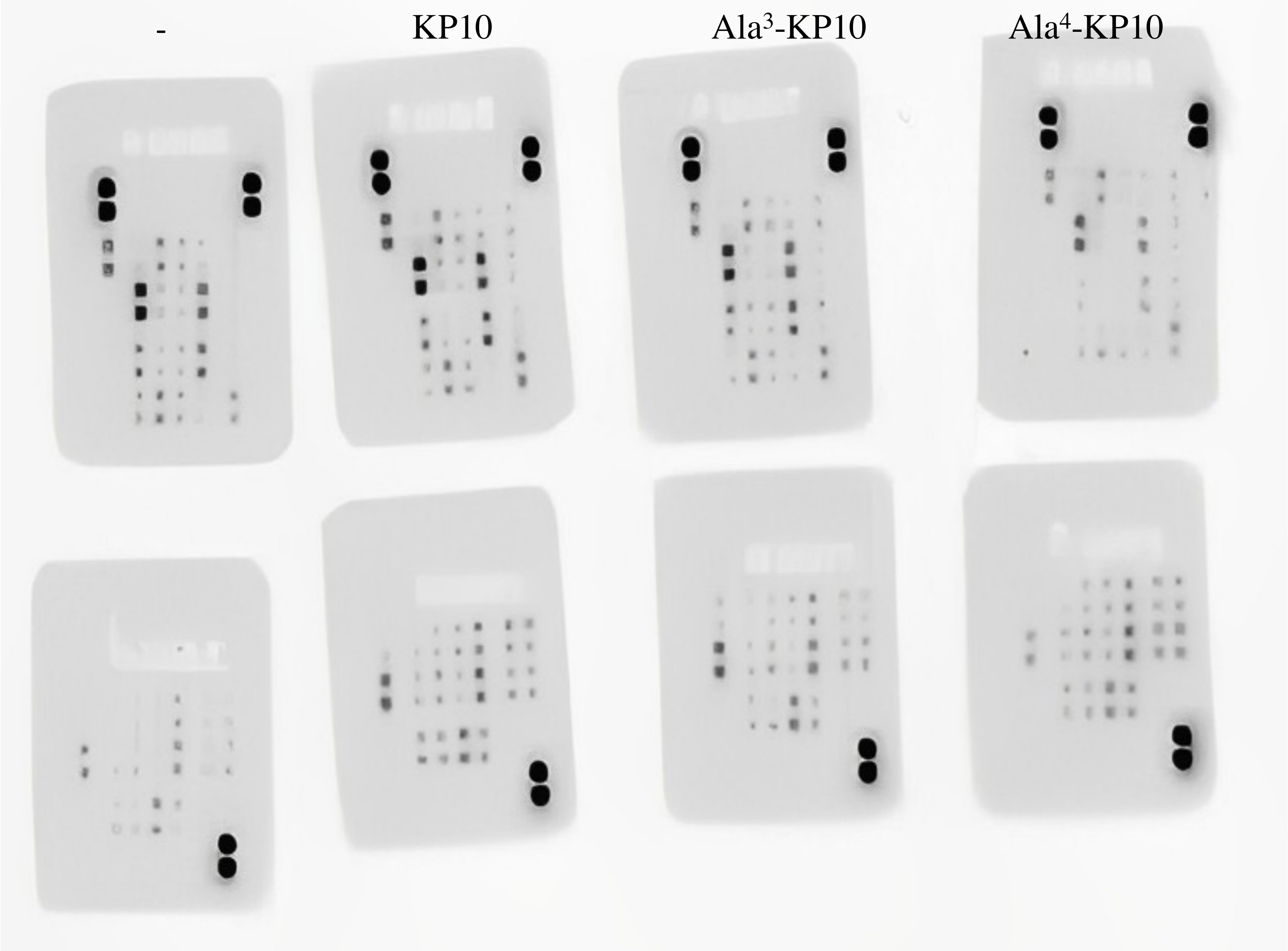

Experiment (n=1) conducted in AGS cells, stimulated with KP10 and analogues.

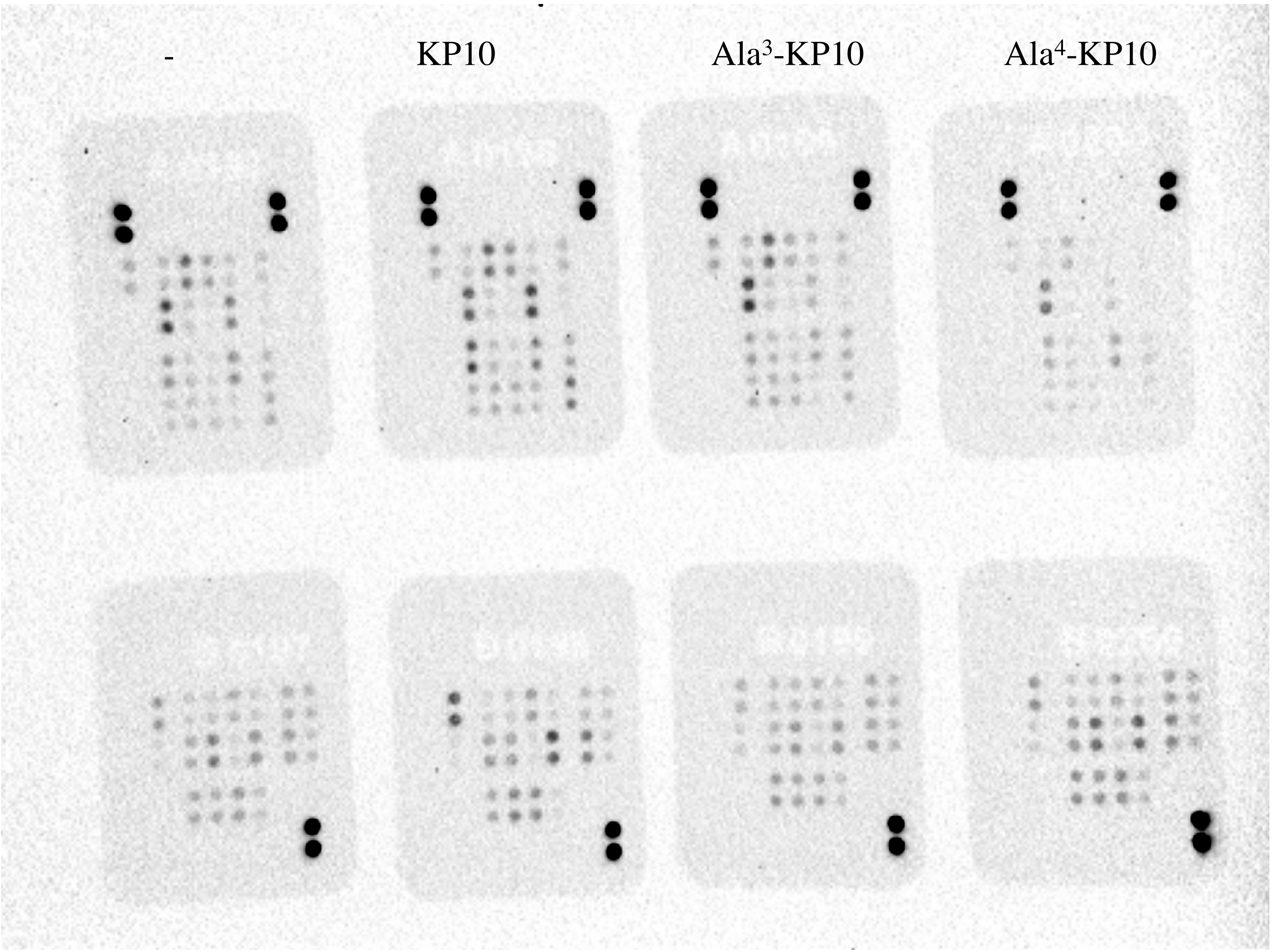

Experiment (n=1) conducted in PC3 cells, stimulated with KP10 and analogues.

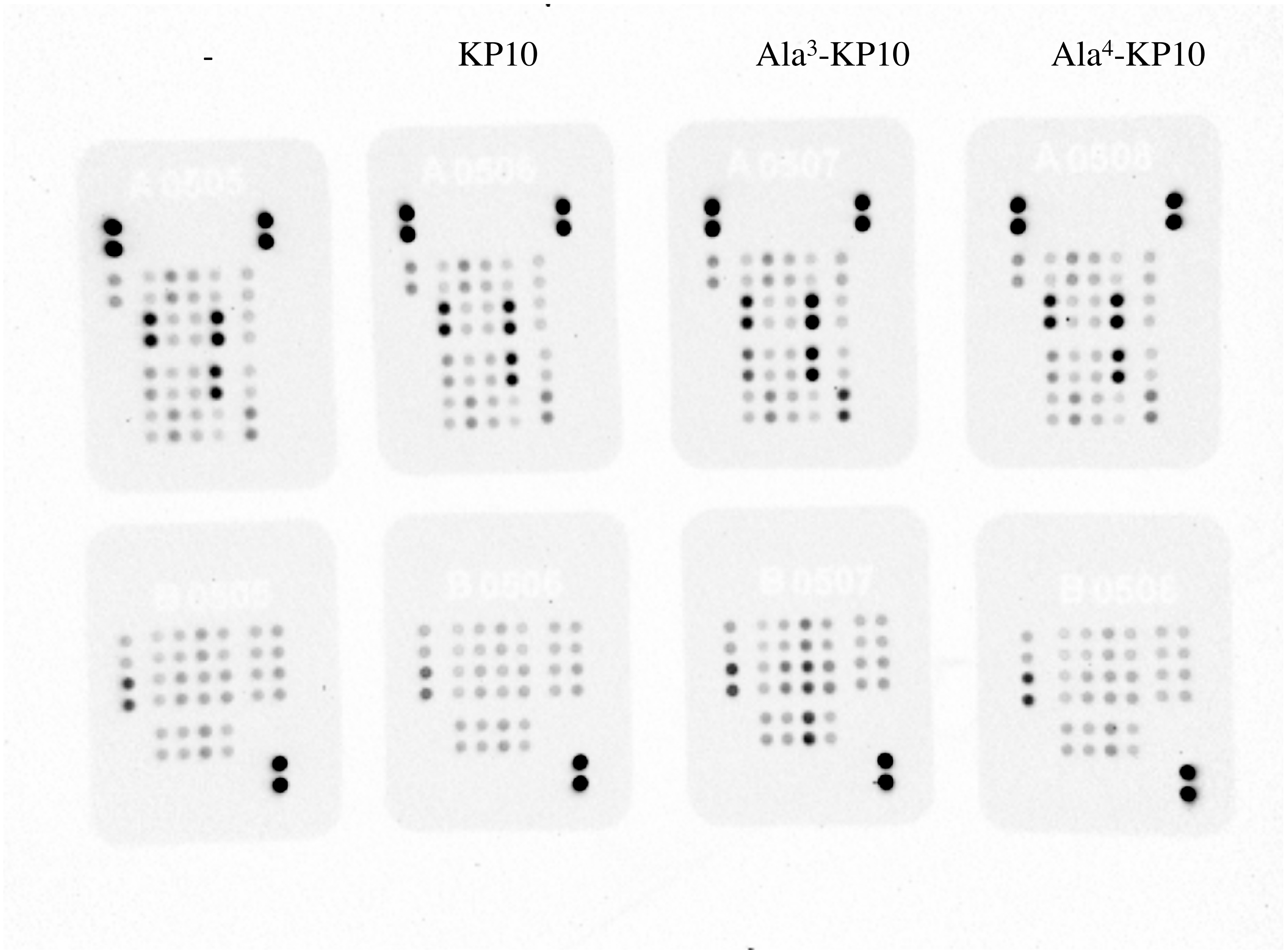

Experiment (n=1) conducted in MCF-7 cells, stimulated with KP10 and analogues.

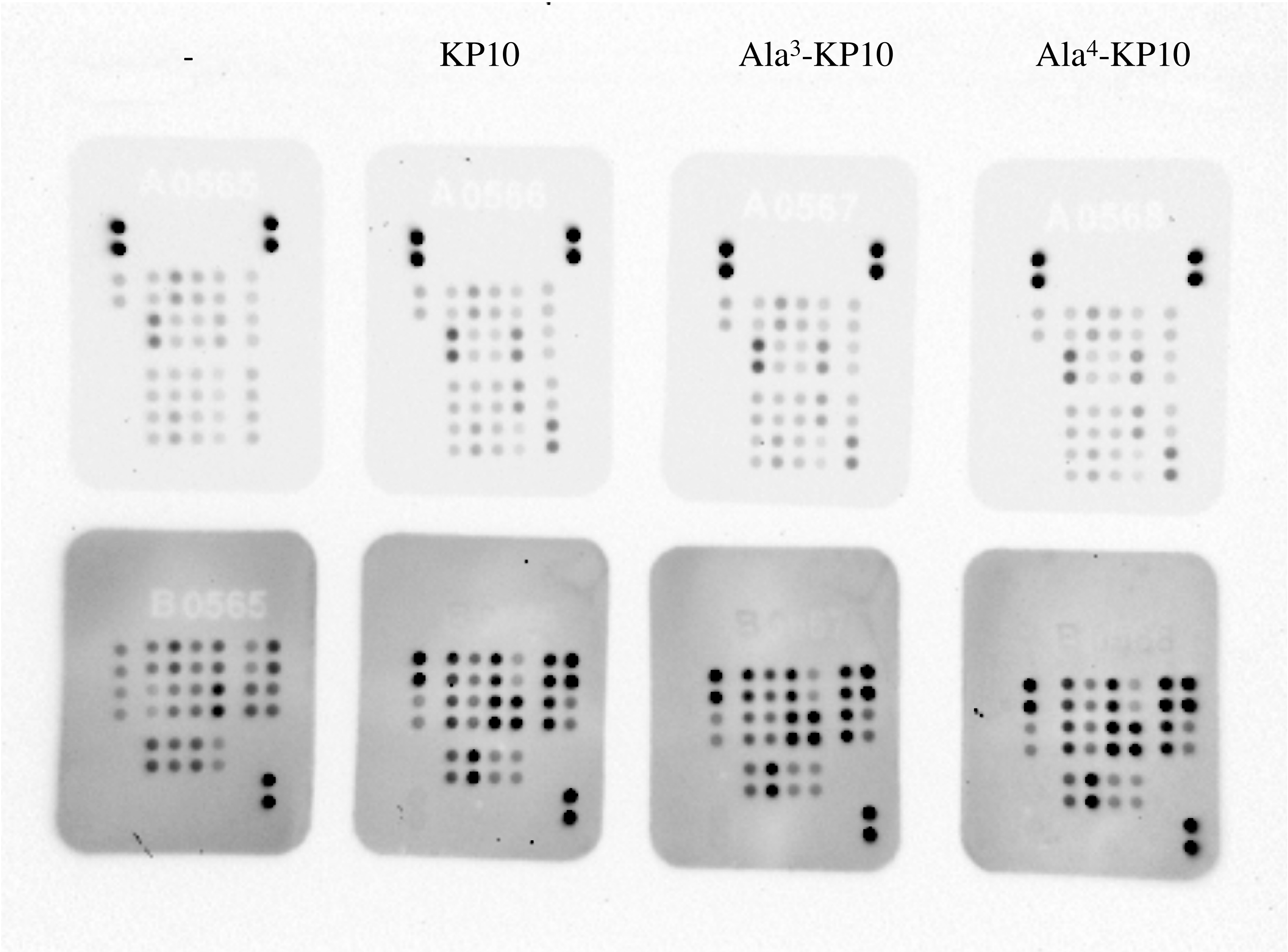

Experiment (n=1) conducted in HeLa cells, stimulated with KP10 and analogues.

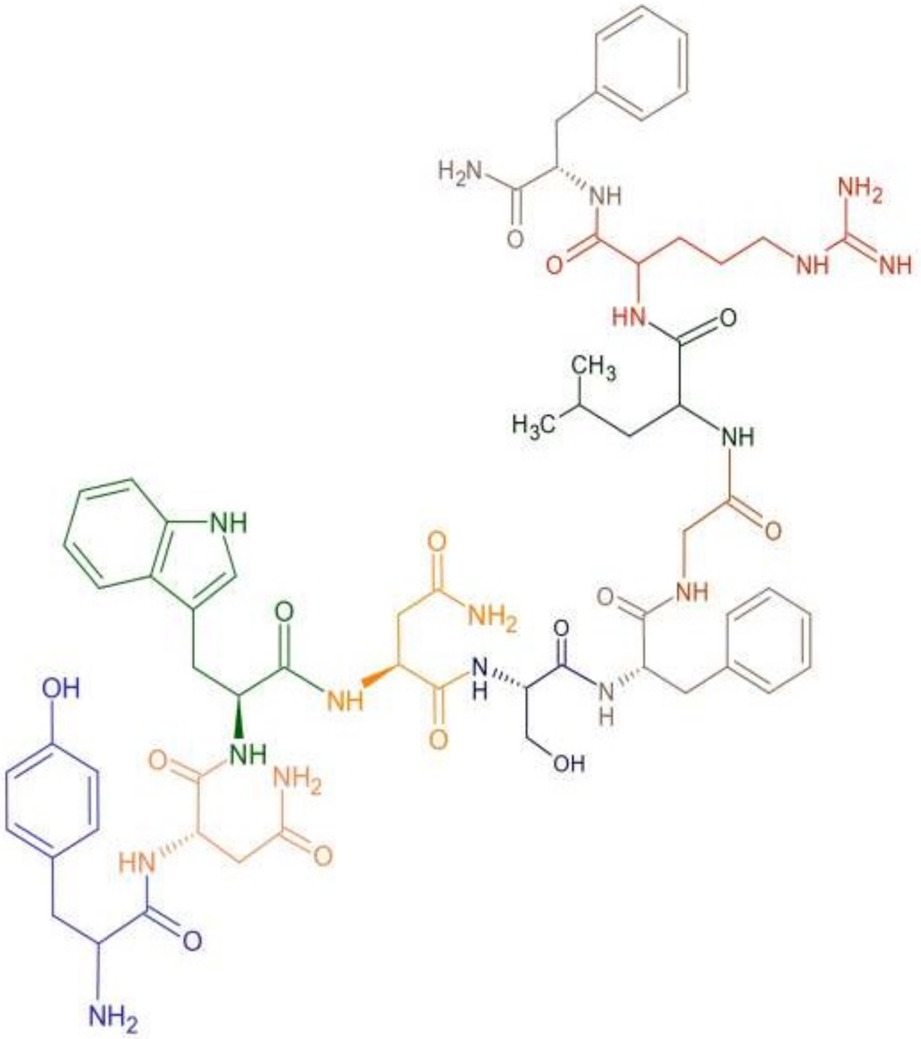

Chemical structure of KP10, the condensed IUPAC system is H-Tyr-Asn-Trp-Asn-Ser-Phe-Gly-Leu-Arg-Phe-NH2.

b) Ala^{4}-KP10

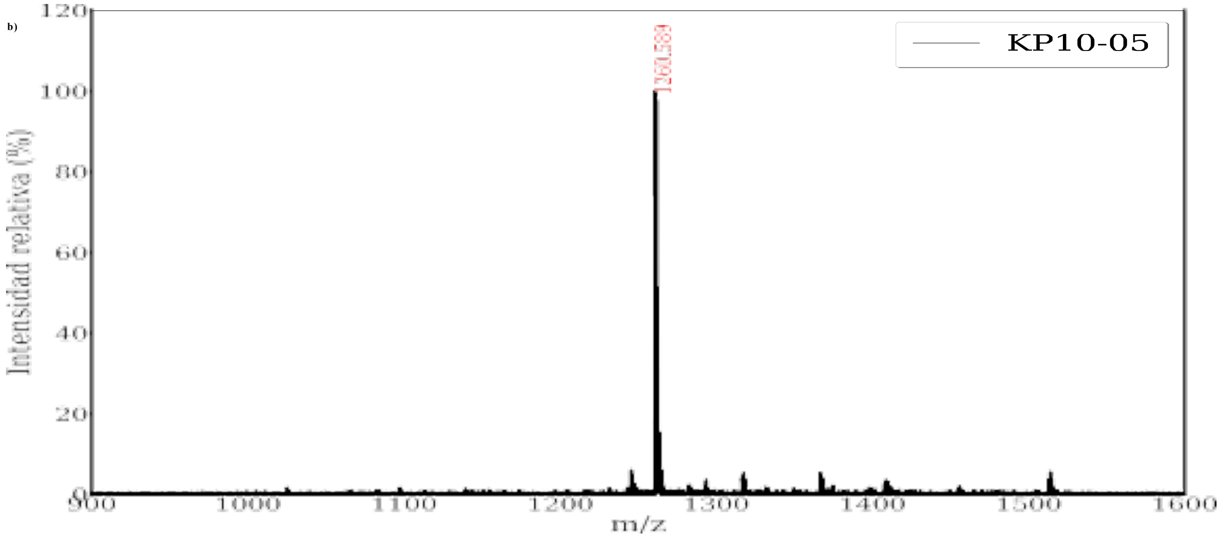

a) Ala^{3}-KP10

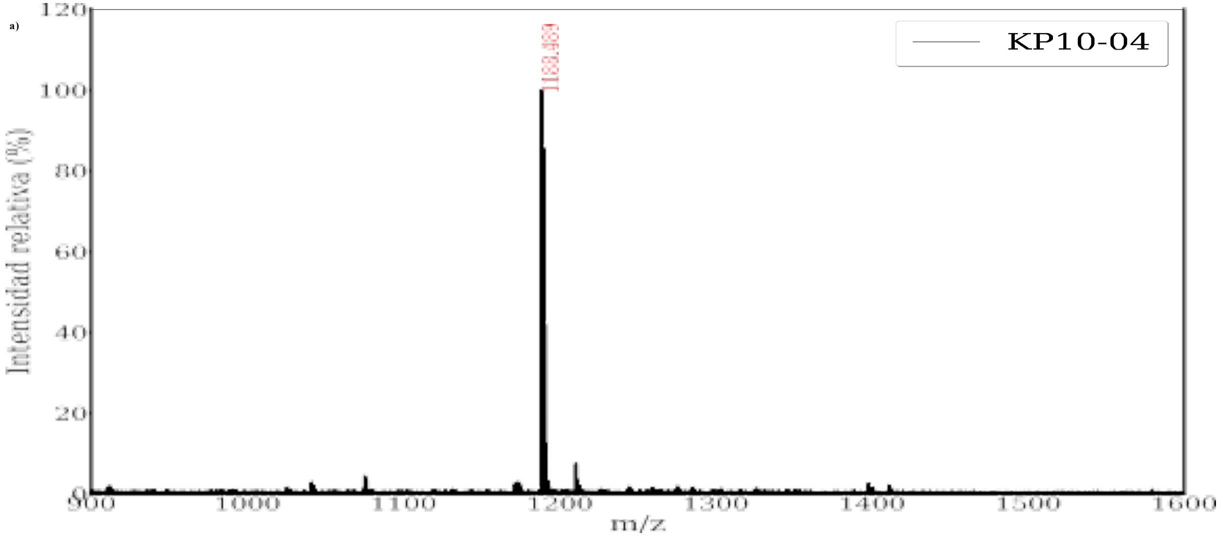

Mass spectra, signals in red indicate the molecular ion of the respective peptide. a) Spectrum of peptide Ala^{3}-KP10. b) Spectrum of peptide Ala^{4}-KP10.
